# "Mushroom cloud": a giant left ventricular pseudoaneurysm after a myocardial infarction due to myocardial bridging – a case report

**DOI:** 10.1186/1476-7120-7-36

**Published:** 2009-07-20

**Authors:** Renata Gomes, Maria João Andrade, Miguel Santos, Sónia Lima, Raquel A Gouveia, Manuel M Ferreira, José Aniceto Silva

**Affiliations:** 1 Department of Cardiology, Hospital Santa Cruz, Av. Prof. Dr. Reynaldo dos Santos, 2795 Carnaxide, Portugal; 2 Department of Cardiothoracic Surgery, Hospital Santa Cruz, Av. Prof. Dr. Reynaldo dos Santos, 2795 Carnaxide, Portugal

## Abstract

Left ventricular pseudoaneurysm is an uncommon complication after transmural myocardial infarction, occurring when a free wall rupture is contained by adhesions of the overlying pericardium preventing acute tamponade. In this report, an unusual case of a 61 year-old male with a giant apical left ventricular pseudoaneurysm after an unnoticed myocardial infarction is presented. On coronary angiogram myocardial bridging of the distal left anterior descending artery was judged to be the infarct related lesion. The echocardiographic diagnosis allowed for a timely surgical intervention which resulted in the patient's full recovery.

## Background

Most left ventricular pseudoaneurysms are caused by myocardial infarction. Other causes are cardiac surgery, trauma and infection. After myocardial infarction, left ventricular pseudoaneurysms are usually located in the inferior and posterior basal walls and rarely in the apex. Although the natural history of acquired left ventricular pseudoaneurysms is not perfectly known, it is accepted that the danger of secondary fatal rupture is high for large or expanding pseudoaneurysms or when the diagnosis is made within the first month after the causal event.

Left ventricular pseudoaneurysms may be accidentally detected by routine imaging techniques or during the investigation of symptoms. Distinction of a pseudoaneurysm from a true aneurysm can be difficult but characteristic imaging features are usually effective in reaching a definitive diagnosis [1-3]. Surgical repair of pseudoaneurysms after myocardial infarction is associated with an important, though acceptable, risk of complications with most patients needing concomitant coronary artery bypasses for significant stenoses [4,5].

We report this case not only because of the unusual features of the left ventricular pseudoaneurym itself (volume, location, "mushroom cloud" appearance), but also because of the rarity of its association with myocardial bridging, the presumed cause of the unnoticed myocardial infarction.

## Case presentation

**Figure 1:**
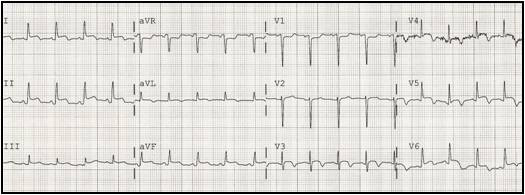
**Twelve-lead ECG at the time of re-admission**. Note ST segment elevation (1.5 mm) in leads I, II, III, AVL, AVF, V3–V6 and T wave inversion from V3 to V6 and in AVL. Note the absence of Q waves.

A 61-year-old black male with no known previous cardiovascular disease was referred from a local hospital for a suspected apical left ventricular pseudoaneurysm. Ten days earlier he had been evaluated at the emergency department for sustained pleuritic chest pain with shortness of breath, a chest X-ray and blood analysis were performed, and he was discharged on antibiotics with a presumptive diagnosis of pneumonia. He was re-admitted with progressive, worsening symptoms and presented with sinus tachycardia (115 beats/min), low blood pressure (82/59 mmHg), a grade 2/6 systolic ejection murmur at the left sternal border and basal rales. The electrocardiogram (Figure 1) exhibited ST segment elevation (1.5 mm) in leads I, II, III, AVL, AVF and V3 to V6, with no pathological Q waves. Cardiomegaly was present on the chest x-ray. Blood tests revealed a mild increase in troponin I (3.7 ng/mL) and a significant elevation of white cell counts and C-reactive protein levels.

**Figure 2:**
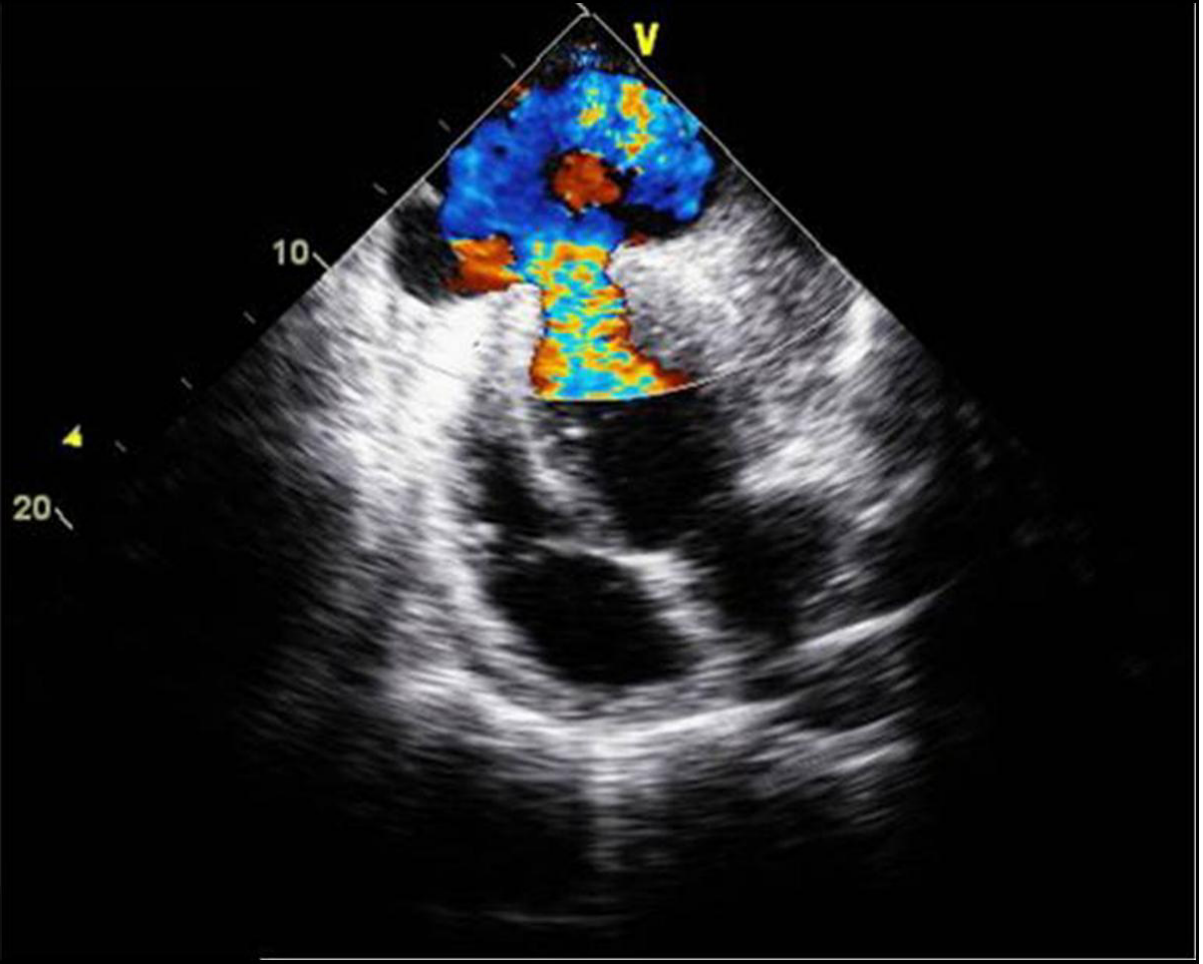
**Transthoracic echocardiography of the apical pseudoaneurysm**. Four-chamber view demonstrates a large discontinuity of the ventricular wall in the apex, with the image resembling a "mushroom cloud".

**Figure 3:**
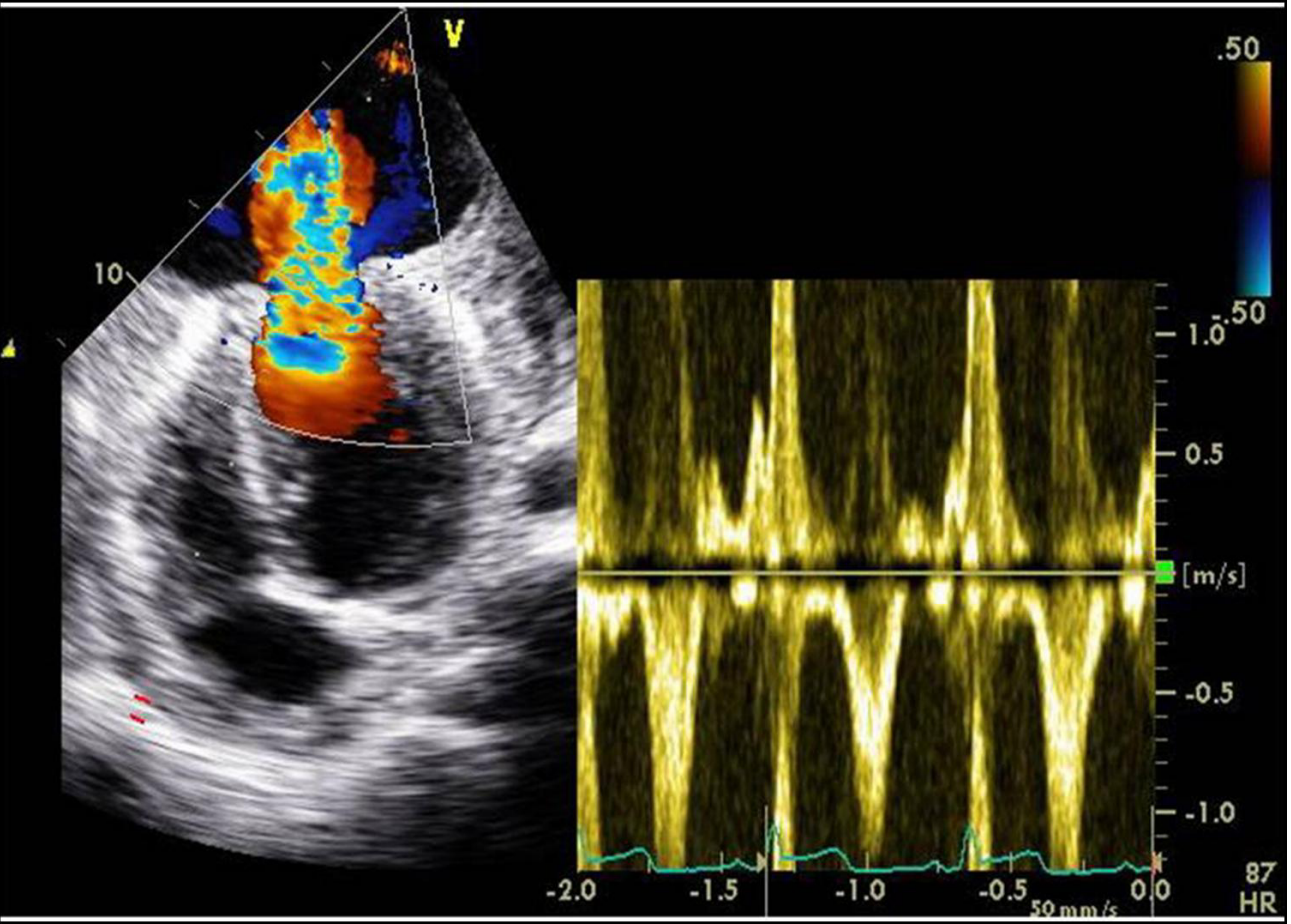
**Continuous-wave Doppler of flow across the neck of the pseudoaneurysm**. Bidirectional turbulent flow into the pseudoaneurysm in systole and away from the pseudoaneurysm, into the left ventricular cavity, in diastole.

**Figure 4:**
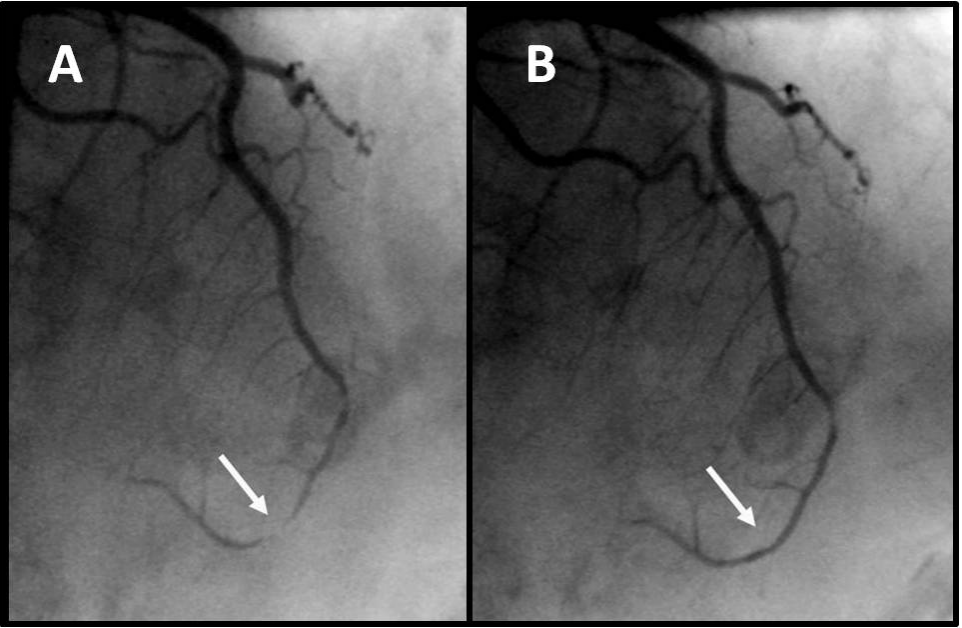
**Demonstration of myocardial bridging**. Significant luminal narrowing of the distal segment of the left anterior descending artery during systole **(A) **and relief during diastole **(B)**.

**Figure 5:**
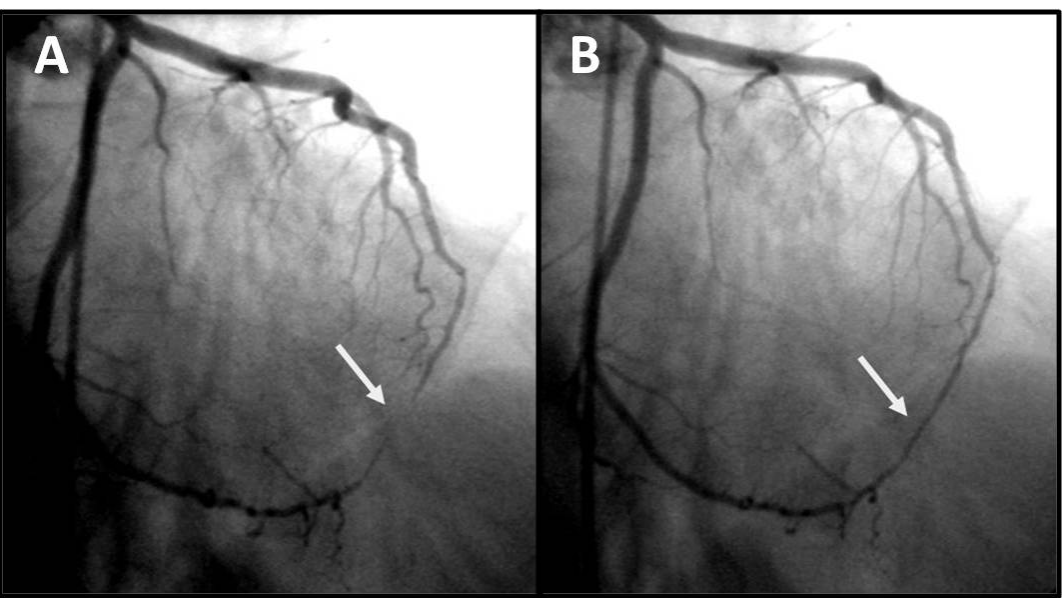
**Coronary angiography of left coronary artery**. Myocardial bridging of the left anterior descending artery during systole (**A**). Absence of constriction during diastole (**B**).

Echocardiography demonstrated an abrupt interruption of the left ventricular myocardial wall in the apex, constituting a narrow (2 cm) necked communication between the left ventricular cavity and a voluminous cavity (10 cm maximum diameter) that corresponded to the pseudoaneurysm (Figure 2; Additional file 1, Additional file 2). With Doppler modalities (colour-flow and continuous-wave), the presence of bidirectional turbulent flow into the pseudoaneurysm in systole and away from the pseudoaneurysm (into the left ventricular cavity) in diastole was observed across the neck of the pseudoaneurysm (Figure 3; Additional file 3). Regardless of the moderate echogenic pericardial effusion, the contour of the pseudoaneurysm wall appeared smooth and regular. From the apical four-chamber view with colour flow Doppler, the image resembled an ominous "mushroom cloud". Urgent coronary angiography was performed before corrective surgery. On the left coronary system, significant phasic systolic vessel compression was observed in the distal segment of the left anterior descending artery, the characteristic "milking" effect of bridging, leading to a cyclic total occlusion of the vessel (Figure 4, Figure 5; Additional file 4, Additional file 5). There were no other angiographically significant coronary lesions.

**Figure 6:**
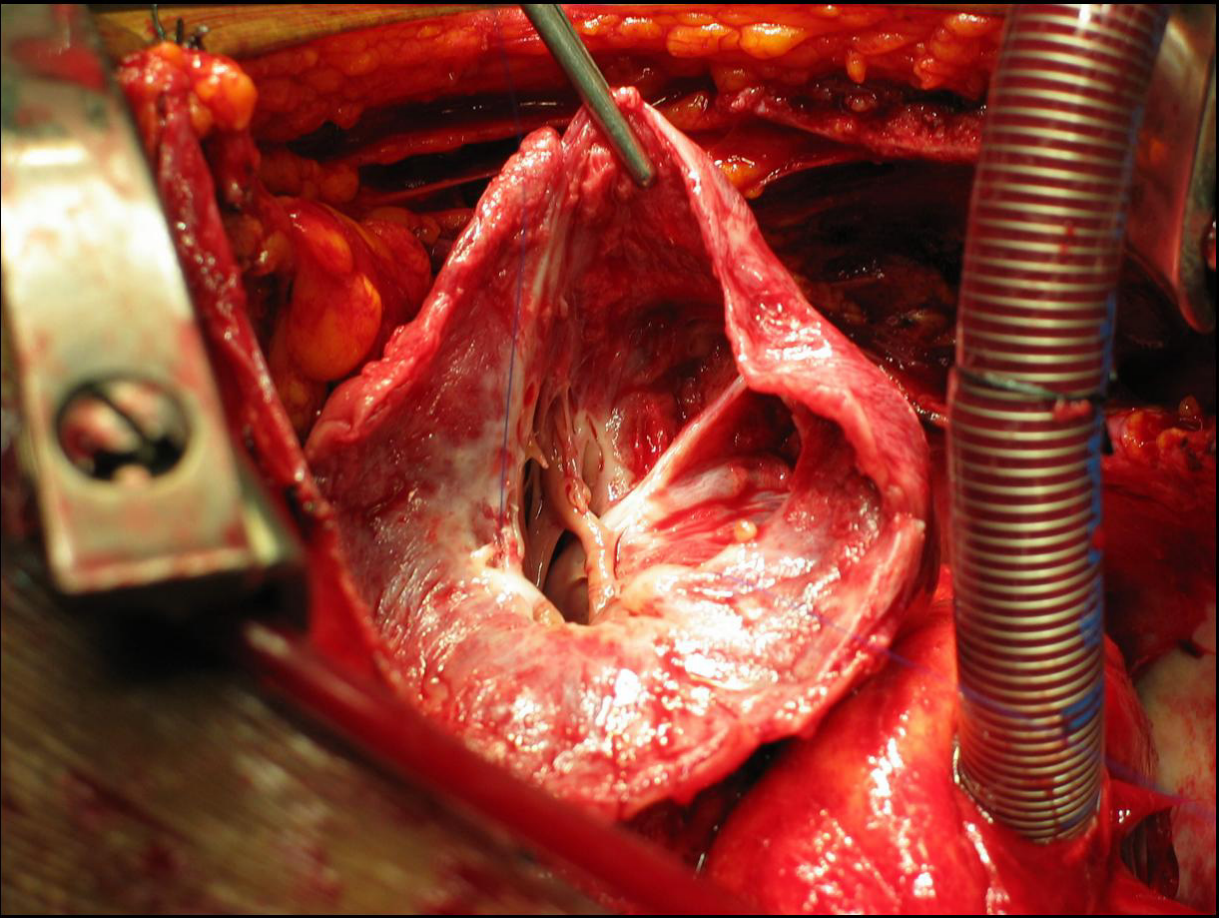
**Intraoperative view of the opened pseudoaneurysm**.

The patient underwent urgent surgical repair using the endoventricular circular patch plasty technique – the "Dor" procedure (Figure 6). The pericardium surrounding the pseudoaneurysm was filled with clots. Histological analysis confirmed the diagnosis of pseudoaneurysm, with the wall of the aneurismal sac composed of fibrous pericardial tissue with no myocardial elements and also revealed evidence of severe pericardial inflammation. The postoperative period was uneventful. Ten months after the operation the patient remains on medical therapy, free of angina and cardiac events.

## Discussion

Though left ventricular pseudoaneurysm formation after myocardial infarction has become a less common event in the era of reperfusion therapy, its diagnosis and management still represents a challenge in the light of its potentially catastrophic consequences. The typical clinical scenario is that of persistent or recurrent symptoms, usually of heart failure, chest pain and dyspnea, after an acute ischemic event [6,7]. In this case, the fact that the myocardial infarction occurred unnoticed made the diagnosis more challenging and precluded the establishment of a time interval between myocardial infarction and the appearance of the pseudoaneurysm.

Echocardiography is a reasonable first diagnostic tool in these cases and, despite the unusual location at the apex, the characteristic imaging features of myocardial wall discontinuity and the presence of a neck smaller than the aneurismal cavity, together with the echogenic pericardial effusion, made it easy to attain a confident diagnosis of pseudoaneurysm, confirmed both during surgery and by the pathologic examination [2].

Most patients with left ventricular pseudoaneurysms after myocardial infarction have severe multivessel disease on preoperative coronary angiography. In a review by Eren and colleagues, of 14 patients who underwent surgery for left ventricular pseudoaneurysm after myocardial infarction, 10 had 3-vessel disease and 4 had 2-vessel disease [4]. In another report from the Cleveland Clinic, Atik and colleagues, in a series of 30 patients who underwent left ventricular pseudoaneurysm repair after myocardial infarction, found only 2 patients without significant coronary lesions [5].

When myocardial infarction occurs in patients with normal coronary arteries it is usually considered secondary to coronary spasm or coronary thrombosis. We found a case report of left ventricular pseudoaneurysm caused by coronary spasm that resulted in a silent myocardial infarction [8].

Myocardial bridging of the coronary arteries, generally considered a benign congenital anomaly, has been reported in association with serious, sometimes fatal cardiovascular events like sudden cardiac death and myocardial infarction [9-11]. Prevalence varies substantially according to the modality and criteria used for detection, being much higher with intravascular ultrasound or at autopsy than with angiography [12]. Myocardial bridges are most commonly located in the left anterior descending artery and although the tunnelled segment is typically spared, the segment proximal to the bridge frequently shows atherosclerotic plaque formation [13].

In recent years, new diagnostic tools such as quantitative coronary angiography, intravascular ultrasound, and intracoronary Doppler flow velocity measurements available in the catheterization laboratory, have greatly contributed to the understanding of the pathophysiologic mechanisms and etiologic factors of ischemic events associated with myocardial bridging. Although the criteria that justify the link between cardiac events and myocardial bridging have not yet been clearly identified, in this patient several factors placed him at an increased risk for cardiac events associated with myocardial bridging, namely the length and severity of diameter reduction of the coronary artery and the presence of delayed persistence of diastolic diameter reduction [14].

## Conclusion

Myocardial bridging is an uncommon clinical aetiology of heart disease. We report the case of a patient with an apical left ventricular pseudoaneurysm with no major obstructions in the epicardial coronary arteries. An obvious myocardial bridging of the distal left anterior descending artery was considered the most likely cause of the myocardial infarction.

## Consent

Written informed consent was obtained from the patient for publication of this case report and accompanying images. A copy of the written consent is available for review by the Editor-in-Chief of this journal.

## Competing interests

The authors declare that they have no competing interests.

## Authors' contributions

RG was responsible for the initial clinical assessment of the patient and diagnostic workup, performed the literature review and wrote the manuscript. MJA performed the bedside echocardiogram, participated in the drafting, and revised the manuscript for important intellectual content. MS and SL participated in the clinical assessment of the patient and diagnostic workup, and revised the manuscript for important intellectual content. RAG revised the document for important intellectual content. MMF performed the surgical procedure. JAS gave final approval to the manuscript. All authors read and approved the final manuscript.

## Supplementary Material

Additional file 1 **2D- Echo parasternal long axis view**. This movie shows a large discontinuity of the left ventricular wall in the apex. Click here for file

Additional file 2 **2D- Echo four-chamber view**. This movie shows an abrupt interruption of the left ventricular myocardial wall in the apex, constituting a narrow communication between the left ventricular cavity and a voluminous cavity that corresponded to the pseudoaneurysm. Click here for file

Additional file 3 **2D- Echo four-chamber view with colour Doppler**. This movie shows the flow across the neck of the pseudoaneurysm. Click here for file

Additional file 4 **Coronary angiography with myocardial bridging**. This movie shows myocardial bridging with significant luminal narrowing of the distal segment of the left anterior descending artery during systole. Click here for file

Additional file 5 **Coronary angiography with myocardial bridging**. This movie shows myocardial bridging with significant luminal narrowing of the distal segment of the left anterior descending artery during systole. Click here for file
